# Mice Lacking M1 and M3 Muscarinic Acetylcholine Receptors Have Impaired Odor Discrimination and Learning

**DOI:** 10.3389/fnsyn.2017.00004

**Published:** 2017-02-02

**Authors:** Wilson Chan, Sanmeet Singh, Taj Keshav, Ramita Dewan, Christian Eberly, Robert Maurer, Alexia Nunez-Parra, Ricardo C. Araneda

**Affiliations:** ^1^Department of Biology, University of MarylandCollege Park, MD, USA; ^2^Instituto de Ciencias Biomédicas, Universidad Autónoma de ChileSantiago, Chile

**Keywords:** olfactory, granule cell, inhibition, olfactory discrimination, adult neurogenesis

## Abstract

The cholinergic system has extensive projections to the olfactory bulb (OB) where it produces a state-dependent regulation of sensory gating. Previous work has shown a prominent role of muscarinic acetylcholine (ACh) receptors (mAChRs) in regulating the excitability of OB neurons, in particular the M1 receptor. Here, we examined the contribution of M1 and M3 mAChR subtypes to olfactory processing using mice with a genetic deletion of these receptors, the M1^−/−^ and the M1/M3^−/−^ knockout (KO) mice. Genetic ablation of the M1 and M3 mAChRs resulted in a significant deficit in odor discrimination of closely related molecules, including stereoisomers. However, the discrimination of dissimilar molecules, social odors (e.g., urine) and novel object recognition was not affected. In addition the KO mice showed impaired learning in an associative odor-learning task, learning to discriminate odors at a slower rate, indicating that both short and long-term memory is disrupted by mAChR dysfunction. Interestingly, the KO mice exhibited decreased olfactory neurogenesis at younger ages, a deficit that was not maintained in older animals. In older animals, the olfactory deficit could be restored by increasing the number of new born neurons integrated into the OB after exposing them to an olfactory enriched environment, suggesting that muscarinic modulation and adult neurogenesis could be two different mechanism used by the olfactory system to improve olfactory processing.

## Introduction

The detection and processing of chemosensory signals by the olfactory system enables a myriad of behaviors, such as food preferences, predator avoidance and social interactions, including keen recognition and mate selection (Sullivan et al., 2015). These odor-triggered behaviors rely on the ability of olfactory circuits to experience short and long-term plasticity, enabling learning and recognition of new odors and the discrimination of salient odor stimuli against a background of less relevant odor cues. The olfactory bulb (OB) plays an important role in early stages of olfactory processing and like other sensory systems, neural activity in the OB is influenced by regulatory feedback from cortical and subcortical areas, including neuromodulatory systems (Fletcher and Chen, 2010; Devore and Linster, 2012). Furthermore, odor discrimination can be improved through odor exposure and this form of perceptual learning involves neuronal plasticity at the level of the main OB (MOB) circuit (Wilson et al., 2004). In the OB, the most abundant neurons are the inhibitory granule cells (GCs), which regulate the excitability of the principal projection neurons, the mitral and tufted cells (MCs) through GABAergic inhibition (Shepherd, 2004). Inhibition of MCs by GCs is thought to contribute to neuronal computations in the OB including sparsening and feature binding of odor representations (Koulakov and Rinberg, 2011; Kato et al., 2012; Lepousez et al., 2013; Gschwend et al., 2015).

The OB receives a rich cholinergic projection from the basal forebrain, specifically the nucleus of the horizontal limb of the diagonal band of Broca (HDB; Zaborszky et al., 1986; Nickell and Shipley, 1988; Kasa et al., 1995). Several studies have indicated that acetylcholine (ACh) regulates the neural circuit of the OB by acting on both nicotinic and muscarinic ACh receptors (mAChRs; Castillo et al., 1999; Doty et al., 1999; Lucas-Meunier et al., 2003; Mandairon et al., 2006a; Prediger et al., 2006; Pressler et al., 2007; Chaudhury et al., 2009; Fletcher and Chen, 2010; Smith and Araneda, 2010; Devore and Linster, 2012; Devore et al., 2012; D’Souza and Vijayaraghavan, 2012; D’Souza et al., 2013; Smith et al., 2015; Bendahmane et al., 2016). The contribution of these receptors to odor perception, however, remains poorly understood. Recent studies have proposed that cholinergic modulation in the MOB enhances olfactory discrimination of odors by sharpening the olfactory receptive field of output neurons, the MCs (Chaudhury et al., 2009; Mandairon et al., 2011; Ma and Luo, 2012). In support of this possibility, several studies have shown that activation of the M1 mAChRs increases the excitability of GCs in the OB (Pressler et al., 2007; Smith and Araneda, 2010; Smith et al., 2015). Thus, M1-mediated activation of GCs could increase inhibition of MCs, which could enhance odor discrimination (Yokoi et al., 1995; Cleland and Linster, 2005; Lepousez and Lledo, 2013).

Interestingly, inhibitory neurons in the OB, including GCs, exhibit adult neurogenesis (Altman and Das, 1965; Lois and Alvarez-Buylla, 1993, 1994), providing a mechanism by which newly replenished neurons contribute to normal circuit function in response to rapidly changing macro and microenvironments (Peretto and Paredes, 2014; Song et al., 2016). Adult neurogenesis has been shown to play an essential role in olfactory-based short-term (Breton-Provencher et al., 2009) and long-term memory (Sultan et al., 2010), perceptual learning (Moreno et al., 2009) and odorant discrimination (Alonso et al., 2006). Importantly, the process of neurogenesis can also be regulated by the cholinergic system (Cooper-Kuhn et al., 2004; Kaneko et al., 2006; Paez-Gonzalez et al., 2014; Asrican et al., 2016), suggesting that it could be a contributing factor to enhance olfactory discrimination.

Here, we study the behavioral consequences of decreased M1 muscarinic cholinergic activity in the OB. To this extent we used two transgenic animal models, mice lacking the M1 and the M1/M3 mAChRs (M1^−/−^ and M1/M3^−/−^ KO mice) and evaluated their olfactory function. These mice showed decreased olfactory discrimination of closely related odors, including stereoisomers and impaired performance in an associative odor-learning task. Interestingly, the M1/M3^−/−^ mice showed an age-dependent reduction in adult neurogenesis of GCs. However, as in the wild type (WT), adult neurogenesis could be stimulated by odor enrichment (OE), which also restored odor discrimination in of the M1/M3^−/−^ mice in the habituation-dishabituation task. These results are in agreement with an essential role of the M1 mAChRs in enhancing olfactory discrimination the OB by modulating GC-mediated inhibition.

## Materials and Methods

### Animals

All experiments were conducted following the guidelines of the IACUC of the University of Maryland, College Park. Experiments were performed in 1–3 months old knockout (KO) female and male mice lacking the M1 or the M1 and M3 mAChR (M1^−/−^ and M1/M3^−/−^ mice, respectively), generously provided by Dr. Jurgen Wess from the NIH. The respective background WT strains of these KO mice are the C57 BL/6 (The Jackson Laboratories) and CF129 mice (Charles River). All mice were obtained from breeding pairs housed in our animal facility, maintained in separate colonies based on strain, and kept on a 12 h light/dark cycle with *ad libitum* access to food and water, unless otherwise indicated.

### Habituation-Dishabituation Test

A clean standard mouse cage (15 cm × 30 cm), without bedding, was used for behavioral testing. Mice were placed in the cage and allowed to familiarize with the test environment for 30 min. During the familiarization phase a wooden cube (2 cm^3^) was placed in the cage in the presence of the mouse. At the conclusion of the familiarization phase, the wooden block was removed for 1 min, and thereafter the mouse was exposed to a wooden block scented with pure water, three times (2 min each), with a 1 min inter-trial interval; these exposures familiarize the mouse to the habituation procedure. The second phase consisted of subsequent exposures to a wooden block scented with 100 μL of the test odor at a 1:1000 dilution (in water) as follows: habituated odor, 3×, dishabituated odor, 1×. Each trial was videotaped and the time the mouse spent investigating the block was quantified offline. The investigation time was determined as the time during which the mouse’s nose was within a 1 cm radius from the block. Unless otherwise indicated, and to minimize differences in behavioral performance, the investigation time in each trial was normalized to the investigation time during the first odor presentation (trial 1). Mice are able to naturally discriminate between the pair of odors when the investigation time is significantly increased during the presentation of the novel odors.

### Associative Odor Learning Test

Mice were housed individually and placed on feed restriction for the whole duration of the associative learning trials, with *ad libitum* access to water. Mice were weighed each day to monitor weight loss and maintained at no less than 85% of their original body weight. For the associative learning task mice were trained to associate an odor with a food reward. A piece of nutter-butter cookie was hidden under a 2 cm^2^ filter paper soaked with 75 μL (1:100 v/v in water) of the associated odor (Figure 2A) both buried under the bedding. A filter paper containing the non-associated odor was buried on the opposite side of the cage, and the cage sides where the filter paper and the reward were hidden was randomized. In these experiments we used the isomers of carvone, which both the CF129 and M1/M3^−/−^ mice failed to discriminate in the habituation-dishabituation paradigm.

**Figure 1:**
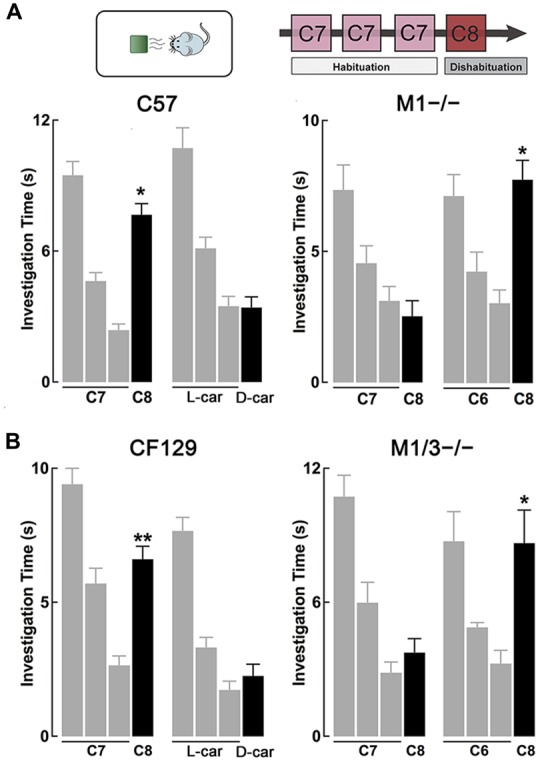
**M1^−/−^ and M1/M3^−/−^ mice exhibit impaired short-term olfactory memory**. **(A)** Top, diagram of the habituation-dishabituation paradigm; during habituation mice are presented three consecutive times with the same odor on a scented wooden block (habituation). On the fourth trial the block is scented with a different odor (dishabituation). Bottom left, wild type (WT) can discriminate an odor pair that differs by one carbon-moiety, ethyl heptanoate (C7) and ethyl octanoate (C8). Three consecutive presentations of C7 (gray bars) resulted in a decrease in investigation time. The ability to discriminate C8 during the fourth trial (black bar) is evidenced by a significant increase in the investigation time (dishabituation, *p < 0.0001). WT mice also habituated to L-carvone (L-car) but failed to dishabituate when presented with D-carvone (D-car). In contrast, the M1^−/−^ mice could not discriminate between C7 and C8, but showed normal investigation times when the odor pair was not chemically similar (*p < 0.001). **(B)** Left, similar to the C57, CF129 WT, discriminated between C7 and C8 (**p < 0.001), but failed to discriminate the carvone stereoisomers. Right, the M1/M3^−/−^ mice exhibited a similar olfactory discrimination deficit as the M1^−/−^ mice, it could discriminate the C6-C8 pair (*p < 0.0001) but not the C7-C8 pair (*P* < 0.4).

**Figure 2:**
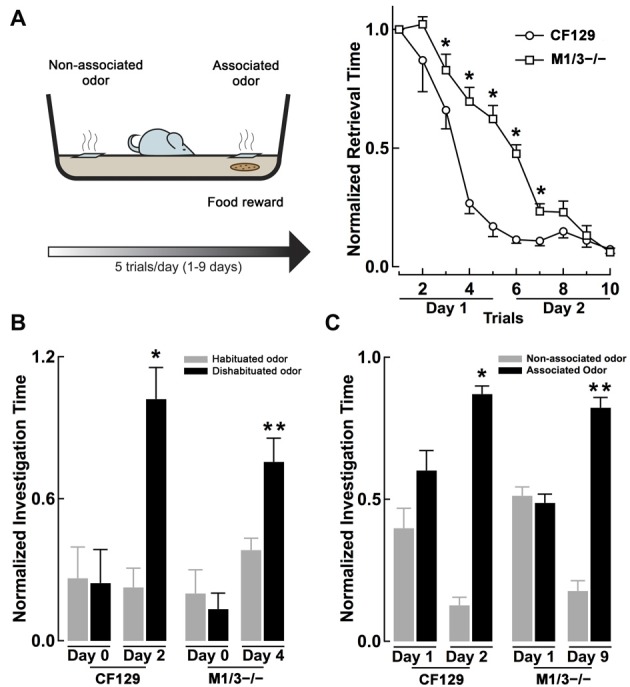
**M1/M3^−/−^ mice exhibit delayed learning in an associative odor learning task. (A)** Left, diagram of the associative learning paradigm, mice were trained to associate an odor with a food reward (a piece of cookie) hidden under a 2 cm square filter paper soaked with L-car (trained odor) for five trials per day. Right, WT CF129 mice (open circles) quickly learned to retrieve the cookie hidden under the filter paper soaked with the odor, as shown by a decrease in retrieval time during each sequential trial. M1/M3^−/−^ mice (open squares) also learned the task but showed a slower learning curve (*p < 0.02). **(B)** At the end of each day of associative learning trials, mice were also tested for their ability to discriminate the stereoisomers in the habituation-dishabituation test (L-car, habituated odor, gray bar; D-car, dishabituated odor, black bar). Before the associative learning trials, both strains are unable to discriminate between the carvone stereoisomers. WT mice were able to discriminate between L and D-car at the end of day two, while the M1/M3^−/−^ mice did after 4 days of associative training (*p < 0.02; ^**^p < 0.006). **(C)** After 2 days of associative learning, WT mice spend significantly more time investigating the associated odor when the carvone isomers are presented simultaneously in the absence of the reward. In contrast, the M1/M3^−/−^ mice spend more time investigating the associated odor only after 9 days of associative learning trials (*p < 0.03, ^**^p < 0.02).

Each day of training consisted of five-trials, in which the time that mice took to retrieve the food reward was recorded. For each animal, the data was normalized to the time the mouse took to retrieve the food reward during the first trial. Under our experimental conditions mice, regardless of the strain, learned the task within 2 days.

At the end of each training day (starting at day 1), mice (CF129 and KO) were tested in the habituation-dishabituation test. In addition, at the end of the day 2 of associative training, mice were presented with both odors simultaneously, in the absence of the reward, for 2 min, and the time they spent sniffing each odor was quantified. For each mouse the time spent investigating each odor was normalized to the total time when both odors are presented together. Mice have learnt to discriminate these odors when they spend a significant greater time investigating the associated odor vs. the non-associated odor. For each strain the associative training was continued until the mice were able to discriminate odors presented simultaneously.

### Response to an Aversive Odor Stimulus and Social Odors

Urine samples were collected from mice using standard protocols (Yang and Crawley, 2009). Evaluation of discrimination of conspecific’s urine was assessed in a similar manner to the procedure described in the habituation-dishabituation paradigm above. Male mice were presented with male urine of the same background strain three times (habituated odor). Following the sequential presentations of the male urine, mice were presented with female urine of the same background strain.

For the aversive odor experiment, mice were consecutively presented for 2 min with either the aversive odor, 2,5-dihydro-2,4,5-trimethylethiazoline (TMT), or a neutral stimulus, water, for 2 min with a 1-min interval between trials. As in the previous experiments, the odor was presented using a wooden cube and was placed on one end of the mouse cage. Odor aversion was quantified based on the amount of time the mouse spends in the corresponding half of the cage in which the odor is presented.

### Visual Discrimination

Visual discrimination was assessed using two odorless, but different colored and shaped Lego® blocks using the habituation-dishabituation paradigm (see above). The Lego® blocks were previously wiped down with 70%-volume based ethanol to remove pre-existing scents. The first object was presented three times sequentially for 2-min with a 1-min inter-trial. The novel object was presented during the fourth trial for 2 min.

### Odor Detection Threshold

The odor detection threshold for the different strains was determined using a modified version of the habituation-dishabituation test. Mice were habituated to three presentations of a wooden block scented with distilled water and on the seventh trial the diluted odor in water was presented (1/60,000, 1/40,000, 1/30,000 and 1/20,000). During these trials the overall investigation time for each mouse decreases, therefore, for each mouse we normalized the investigation time for each trial to the first trial (water).

### Quantification of Adult Neurogenesis

To quantify adult neurogenesis we used 5-bromo-2′-deoxyuridine (BrdU), 10 mg/mL in phosphate-buffered saline (PBS). One-month old mice received an intraperitoneal injection of BrdU (100 mg/kg), 3× every 2 h. After the injections, mice were kept in the animal facility with food and water *ad libitum* for 1 month. Mice were sacrificed and perfused intracardially with 10 mL of cold 1× PBS, followed by 25 mL of cold 4% paraformaldehyde (PFA; Electron Microscopy Services). Brains were then excised and post-fixed in 4% PFA for 4 h at 4°C, and then immersed in 30% sucrose (Fischer) diluted in 1× PBS overnight at 4°C. Following fixation, the brains were sectioned into 20 μm sections using a cryostat and immunohistochemically stained for BrdU.

Staining required an initial stage of antigen retrieval where slices were incubated in 2 M HCl at 37°C for 1 h. This was followed by a blocking period of 2 h where slices were incubated in 10% donkey serum in PBS with 0.1% Triton X-100 (PBS-T). Slices were then incubated overnight in 1:150 dilutions of rat anti-BrdU (Abcam, ab6325) and mouse anti-NeuN (Chemicon International, MAB377) antibodies in PBS-T containing 2.5% donkey serum. The following day, slices were incubated for 2 h in a secondary solution containing 1:750 donkey anti-rat (Invitrogen, A-21209) and donkey anti-mouse (Invitrogen, A-21202) antibodies, conjugated with Alexa594 and Alexa488 fluorophores, respectively. Secondary antibody dilutions were made in PBS-T containing 2.5% donkey serum. To assess basal level of neurogenesis in both CF129 (WT) and M1/M3^−/−^ strains, mice were injected with BrdU at postnatal week (PW) 8 or PW14 and then sacrificed at PW12 or PW18, respectively. For BrdU positive neuron quantification, we used stereology for unbiased cell counting described in detailed in Nunez-Parra et al. (2011). For OE (see below), BrdU injection was performed at PW14 and the animals sacrificed at PW18. These mice were tested in the habituation-dishabituation before, and after injection of BrdU followed by OE.

### Odor Enrichment

Odor-enriched mice were exposed daily, for 24 h, to different aromatic fragrances placed in a metal tea ball hanging from the cover of standard breeding cages. Control mice were reared under the same conditions except that the tea ball was left empty. OE occurred over a 40-day period. Aromatic fragrances included lavender, garlic, paprika, marjoram, curry, rosemary, nutmeg, thyme, basil leaves, cumin, cardamom, tarragon, whole cloves, chocolate, celery, anise, ginger, lemon, orange and banana. Mice were exposed to each fragrance once over the enrichment period (Rochefort et al., 2002).

### Odor Stimuli and Data Analysis

We used urine samples from mature and sexually naïve, male and female, mice. A gentle pressure on the mice’ lower back was used to induce urine voiding (Watts, 1971). Collected urine was then pooled by sex and strain and stored at −20°C until needed. Odors used for the habituation-dishabituation and hidden-cookie were obtained from Sigma-Aldrich (St Louis), at the purest grade: Butanol (C4-OH), Pentanol (C5-OH), Ethyl hexanoate (C6), Ethyl heptanoate (C7), Ethyl octanoate (C8), L- Carvone (L-car), D-Carvone (D-car); (+)-Limonene (+)-lim, (−)-Limonene (−)-lim. TMT was obtained from (PheroTech). Odor dilutions were made in water on the day of the experiment. Unless otherwise stated, all comparisons made were evaluated using a Student’s *t*-test to determine statistically significant differences.

Due to the longitudinal nature of the experiments conducted, we used a paired *t*-test for determining statistical significance. In addition, we used the GPower software^1^ to verify the statistical power for our *t*-tests. Using this software, we found that our reported significant differences had a power level of 99%–99.99%. The sample size for each experiment ranged from 4 to 23 mice and we only used males for our experiments to avoid incorporating an additional variable (estrous cycle and hormonal fluctuations). All mice were used for one test only, unless otherwise stated (i.e., associative learning). The error bars correspond to the standard error of the mean (SEM).

## Results

### M1^−/−^ and M1/M3^−/−^ Mice Exhibit Disrupted Short-Term Memory

Previous work has shown that cholinergic modulation of excitation of GCs in the OB occurs mainly through activation of type M1 mAChRs (Pressler et al., 2007; Smith and Araneda, 2010; Smith et al., 2015), however, the contribution of this receptor to odor perception remains unknown. To test the ability of the M1^−/−^ KO mice to naturally discriminate structurally similar odors, we used the habituation-dishabituation paradigm. We first characterized the behavior of the WT background strain of the M1^−/−^ KO, the C57/BL6 mice (C57). As shown in Figure 1A, bottom left, WT mice habituated to sequential presentations of an odor-scented wooden block (see “Materials and Methods” Section), and showed a decrease in the investigation time. The investigation time for the C7 ester decreased by 75%, across three odor presentations (trial 1, 9.5 ± 0.6 s; trial 3, 2.4 ± 0.3 s; *n* = 28; *p* < 0.0001). WT mice readily discriminated structurally linear esters that differed by one carbon moiety, and accordingly the investigation time increased during the presentation of the C8 ester (trial 4, 7.7 ± 0.5 s; *p* < 0.0001). In addition, WT mice discriminated the C5/C6 ester pair and esters that differed by two carbons (Table 1).

**Table 1:** **Table showing the performance of wild type (WT) and M1^−/−^ mice in the habituation-dishabituation (H/D) for different odor pairs, aversive odor, novel object recognition and social odor preference**.

Mouse strain	H/D Odor pair	Investigation time habituation (trial 3)	Investigation time dishabituation	*p* value	*n*
WT	C6/C5	1.2 ± 0.6 s	6.3 ± 1.7 s	*p* < 0.03	4
WT	C4-OH/C5-OH	2.9 ± 0.7 s	9.2 ± 1.0 s	*p* < 0.0002	10
WT	(+)-lim/(−)-lim	2.6 ± 0.6 s	2.7 ± 0.3 s	*p* > 0.9	4
M1M1^−/−^	L-car/D-car	2.6 ± 0.9 s	2.9 ± 0.7 s	*p* > 0.3	4
	Reference vs. test odor	Normalized investigation time, (reference odor)	Normalized investigation time (test odor)		
WT	H_2_O/TMT	0.63 ± 0.05	0.46 ± 0.07	*p* < 0.05	4
M1M1^−/−^	H_2_O/TMT	0.59 ± 0.03	0.41 ± 0.03	*p* < 0.01	4
WT	H_2_O/Female urine	0.30 ± 0.05	1.16 ± 0.08	*p* < 0.01	4
M1M1^−/−^	H_2_O/Female urine	0.35 ± 0.06	1.03 ± 0.16	*p* < 0.01	4
	H/D Visual Pair	Normalized investigation time, Lego 1	Normalized investigation time, Lego 2		
WT	Lego 1 vs. Lego 2	0.43 ± 0.05	0.67 ± 0.06	*p* < 0.05	8
M1M1^−/−^	Lego 1 vs. Lego 2	0.33 ± 0.07	0.83 ± 0.07	*p* < 0.01	8

However, although the WT mice habituated to the “L” isomer of carvone (L-car, trial 1, 10.7 ± 0.5 s; trial 3, 3.5 ± 0.4 s; *n* = 23; *p* < 0.0001), the investigation time did not significantly increase during the presentation of D-carvone (D-car, 3.4 ± 0.5 s, *p* > 0.5), suggesting that the WT are not able to discriminate the “L” and “D” enantiomers. This natural inability to discriminate odors extended to other structurally related molecules such as the C4/C5 alcohol pair and the enantiomers of limonene (Table 1).

Similar to the WT mice, M1^−/−^ mice engaged normally in the habituation phase of the test and exposure to C7 resulted in a 58% decrease by the third odor presentation (Figure 1A, bottom right). In contrast, unlike the WT, the M1^−/−^ mice failed to discriminate esters whose structure differed by one carbon moiety. The investigation time for the habituated odor (C8) was not different to the response to the habituated C7 (C7, 3.1 ± 0.6 vs. C8; 2.5 ± 0.6 s; *n* = 8; *p* > 0.4; Figure 1A, bottom right). Similar results were obtained with the C5/C6 ester pair, the C4/C5 alcohol pair, and the stereoisomers of Limonene and the “L” and “D” carvone enantiomers (Table 1).

In contrast, the M1^−/−^ mice could readily discriminate between C6 and C8, an ester pair whose structure differs by two carbons (C6, 3.1 ± 0.5 s; C8, 7.8 ± 0.7 s; *n* = 10; *p* < 0.001; Figure 1A, bottom right). In summary, this data indicates that ablation of the M1 mAChR does not affect odor habituation *per se* and that muscarinic neuromodulation trough M1 mAChR is not required to discriminate between perceptually different odor molecules. However, olfactory discrimination of structurally similar molecules is impaired in the M1^−/−^ mice.

Surprisingly, in electrophysiological recordings of the accessory OB (AOB), the OB region that processes pheromonal information, we found that GCs still exhibited an excitatory muscarinic response in the M1^−/−^ mice (not shown). Further pharmacological characterization indicated that the depolarization in AOB was sensitive to M3 mAChRs blockers, suggesting an up-regulation of these receptors in the M1^−/−^ KO mice (see also Smith et al., 2015). To circumvent this problem, for the next experiments we determined the contribution of M1-mAChRs to olfactory processing using a mouse that lacks both the M1 and M3 receptors (M1/M3^−/−^ double KO mice). In parallel, we performed the same battery of experimental behavioral paradigms in the M1^−/−^ for comparison purposes.

As shown in Figure 1B, left, CF129 mice (the WT strain from which the M1/M3^−/−^ derives) showed robust habituation during the sequential presentations of C7 (trial 1, 9.4 ± 0.6 s; trial 3, 2.6 ± 0.4 s; total decrease 72%; *n* = 20; *p* < 0.001) and discriminated the C8 ester (trial 4, 6.6 ± 0.5 s; *p* < 0.001). Although the CF129 mice habituated to L-car (trial 1, 7.7 ± 0.5 s; trial 3, 1.7 ± 0.3 s; *n* = 16; *p* < 0.001), the investigation time did not significantly increase during the presentation of D-car (trial 4, 2.3 ± 0.4 s; *p* > 0.3), suggesting that the CF129 mice and the C57 exhibited a similar olfactory discrimination profile.

Similar to the WT mice, M1/M3^−/−^ mice was engaged normally in the habituation-dishabituation test during the presentation of the C6. The M1/M3^−/−^ readily discriminated between a pair of ethyl esters that differed by two carbons, C6/C8 (C6 and C8; trial 1, 10.9 ± 1.7 s vs. trial 3, 4.1 ± 0.7 s; total decrease 63%; *n* = 14; *p* < 0.05; Figure 1B, right). In contrast, unlike the WT and mirroring the M1^−/−^ behavior, the M1/M3^−/−^ mice failed to discriminate esters whose structure differed by one carbon moiety. As shown in Figure 1B, right, the investigation time for the novel odor (C8) was not different to the response to the habituated odor (trial 1, 2.8 ± 0.5 vs. trial; 3.7 ± 0.6 s; *n* = 20; *p* > 0.6). Also, like the WT, they failed to discriminate the “L” and “D” carvone enantiomers (see Figure 2B). We also tested the detection threshold in the M1/M3^−/−^ mice using a modified version of the habituation-dishabituation paradigm (see “Materials and Methods” Section). The odor detection threshold for the C7 in the M1/M3^−/−^ mice was the same as in the WT, with detection of the odor occurring at 1/30,000. Thus, for the CF129 at 1/40,000 the investigation time was not different between water and the diluted odor (0.35 ± 0.03 vs. 0.37 ± 0.03, *n* = 4, *p* > 0.07) but significantly increased when the odor was presented at the 1/30,000 dilution (0.24 ± 0.04 vs. 0.75 ± 0.01, *n* = 4, *p* < 0.02). Similarly for the M1M3^−/−^, the investigation of water and the diluted odor was not different (0.31 ± 0.03 vs. 0.42 ± 0.04, *n* = 3, *p* > 0.08) but significantly increased when the odor was presented at the 1/30,000 dilution (0.22 ± 0.04 vs. 4 0.64 ± 0.03, *n* = 3, *p* < 0.01).

### Associative Odor Learning is Disrupted in the M1/M3^−/−^ Mice

Muscarinic receptors are known to play a role in associative olfactory memories (De Rosa and Hasselmo, 2000; Saar et al., 2001), not only at the level of the OB, but also in the olfactory cortex (PC), one of the main targets of the OB output neurons (Nagayama et al., 2010). Therefore, we examined whether cholinergic deficits had an effect on long-term memory, specifically odor recognition elicited by an associative-odor learning paradigm. It has been shown that repetitive exposure of a non-discriminant odorant results in an increase of the rodent ability to discriminate them and that this improvement depends on activation of mAChR (Fletcher and Wilson, 2002). Thus, we compared the ability of the CF129 and M1/M3^−/−^ mice to discriminate the isomers of carvone, after associative training with a food reward. For this, mice were trained to associate L-car with a cookie reward and D-car was used as the non-associated odor (see “Materials and Methods” Section, Figure 2A left). As shown in Figure 2A right, by the end of the second day of training both the WT and M1/M3^−/−^ mice learned the task; however, during training the learning curve for the M1/M3^−/−^ mice was significantly slower than the WT mice. By the end of the first day of training, the M1/M3^−/−^ mice take a significantly longer time to find the food reward than the WT (normalized retrieval time, 0.66 ± 0.06 vs. 0.17 ± 0.04; *n* = 4; *p* < 0.004). Similar results were obtained with the M1^−/−^ mice (normalized retrieval time, 0.94 ± 0.07 vs. 0.17 ± 0.04, *n* = 4, *p* < 0.05).

To test the ability of the mice to discriminate between the associated and non-associated odors, each odor was presented on a different side of the cage in the absence of the reward. We found that WT but not M1/M3^−/−^ mice were able discriminate between the carvone isomers by the end of the second day of training (L-car, 0.87 ± 0.03 s; D-car, 0.13 ± 0.03 s; *n* = 4; *p* < 0.01; Figure 2C). In contrast, the M1/M3^−/−^ mice required a total of 9 days of associative learning trials before they could perform successfully in this task (L-car, 0.82 ± 0.04 vs. D-car; 0.18 ± 0.04; *n* = 4; *p* < 0.05; Figure 2C). Experiments performed with the M1^−/−^ showed a similar pattern requiring 8 days of associative training to discriminate the isomers (L-car, 0.77 ± 0.05 vs. D-car 0.23 ± 0.05; *n* = 4, *p* < 0.05).

In addition, at the end of each training day we examined the performance of the mice in the habituation-dishabituation test (L-car was used as the habituated odor and D-car as the dishabituated odor). As shown in Figure 2B, by the end of the second day of training, the WT but not the M1/M3^−/−^ mice were able to discriminate the stereoisomers of carvone (WT, L-car, 0.24 ± 0.05; D-car, 0.82 ± 0.11; *n* = 4; *p* < 0.05: M1/M3^−/−^, L-car, 0.34 ± 0.07; D-car, 0.42 ± 0.13; *n* = 4, *p* < 0.5). However, after 2 additional days of associative training the M1/M3^−/−^ mice were able to discriminate the carvone isomers (L-car, 0.29 ± 0.07; D-car, 0.77 ± 0.03; *n* = 4; *p* < 0.05; Figure 2B). Therefore, in addition to their difficulty in naturally discriminating structurally similar odors, the M1/M3^−/−^ mice show impaired associative learning capability showing a much slower learning rate than WT mice.

### M1/M3^−/−^ Exhibit Normal Olfactory-Mediated Behaviors When Exposed to Complex Social Odors

**Figure 3:**
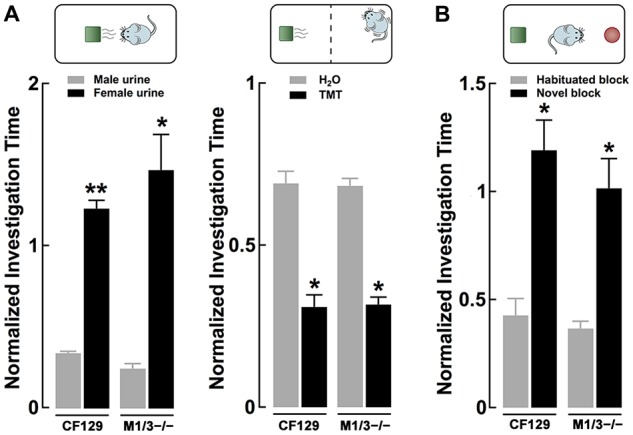
**Sensory deficits in the M1/M3^−/−^ mice do not translate to social odors**. **(A)** Left, discrimination of urine, a complex social odor, is normal in the KO mice. Both M1/M3^−/−^and WT male mice habituated to same sex conspecific urine and presentation of the opposite sex’s urine resulted in a significant increase in investigation time (**p < 0.01; *p < 0.02). Right, the response to naturally aversive odor, 2,5-dihydro-2,4,5-trimethylethiazoline (TMT), was normal in the M1/M3^−/−^ mice. The aversive response was measured as the time the animals spent at the opposite side of the cage where a TMT (or water) “scented” block was placed. Both WT and M1/M3^−/−^ mice spend less time on the side of the cage with TMT (*p < 0.02). **(B)** Like the WT, M1/M3^−/−^ mice have normal visual discrimination and show increased investigation time in the presence of a novel object (*p < 0.02).

Odors found in nature are complex mixtures of odorants, which animals use to elicit social behaviors critical for survival and reproduction. Therefore, we tested the ability of these mice to engage in the investigation of a more complex odor mixture; for this we used urine, which is a social odor containing several semiochemicals (Tirindelli et al., 2009). As seen in Figure 3A left, both WT and M1/M3^−/−^ mice habituated to consecutive presentations of male urine and increased the investigation time in the presence of the novel odor (female urine; CF29, 0.34 ± 0.01 vs. 1.23 ± 0.05; *n* = 4; *p* < 0.01; M1/M3^−/−^ 0.24 ± 0.03 vs. 1.47 ± 0.21; *n* = 4; *p* < 0.02). We also exposed the animals to an innate aversive odor, TMT, which is a component of fox feces, a natural mice predator that elicits a variety of fear-like behaviors in rodents (Rosen et al., 2015). Similarly, the response to an aversive odor was not different between the WT and the M1/M3^−/−^ mice (CF29, 0.69 ± 0.04 vs. 0.31 ± 0.04; *n* = 4; *p* < 0.01; M1/M3^−/−^ 0.68 ± 0.02 vs. 0.32 ± 0.02; *n* = 4; *p* < 0.02; Figure 3A right). Furthermore, to control for the possibility that our mouse model could have impaired vision, we exposed them to legos of different colors (see “Materials and Methods”). We found that M1/M3^−/−^ mice are not different than WT (CF29, 0.34 ± 0.01 vs. 1.23 ± 0.05; *n* = 4; *p* < 0.01: M1/M3^−/−^ 0.24 ± 0.03 vs. 1.47 ± 0.21; *n* = 4; *p* < 0.02; Figure 3B). For comparison purposes we tested the same odors and novel objects in the M1^−/−^ and found a normal sensory discrimination profile (Table 1).

### M1/M3^−/−^ Mice Exhibit an Age Dependent Decrease in Adult Neurogenesis

**Figure 4:**
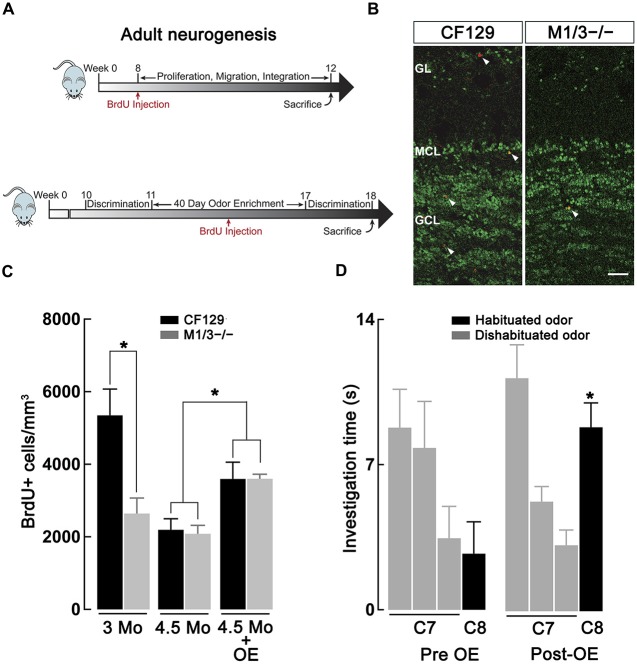
**Adult neurogenesis in the olfactory bulb (OB) is decreased in the M1/M3^−/−^ mice. (A)** Diagram showing the experimental design used to quantify basal adult neurogenesis. Top, male WT CF129 and M1M3 ^−/−^ mice were injected with BrdU at 2 months of age and sacrificed 1 month later (see “Materials and Methods” Section). Bottom, for the odor enrichment (OE) experiments mice were exposed to different odors for 40 days, injected with BrdU 20 days within the OE period and sacrificed at week 18. Mice were tested in the habituation-dishabituation paradigm before and after OE. **(B)** Confocal images of adult main OB (MOB) sections of WT (left) M1M3^−/−^ mice (right) double stained for BrdU (red) and NeuN (green); white arrow heads indicated the presence of BrdU+ positive cells. The glomerular layer (GL) is characterized by a low density NeuN staining in the upper portion of each image, while the granule cell layer (GCL) is represented by the higher density staining in the lower portion of each image. Calibration bar is 50 μm. **(C)** A quantification of the density of adult-born neurons reveals significantly diminished levels of BrdU+ cells in the M1/M3^−/−^ mice compared to the WT mice at 3 months of age (*p < 0.05), but not at 4.5 months of age. After OE both strains show an increase in adult neurogenesis (*p < 0.05). **(D)** After OE the M1/M3^−/−^ mice show a significant increase in the investigation of the dishabituated odor during tests using the C7-C8 odor pair (*p < 0.001).

Previous studies have indicated a link between cholinergic modulation and adult neurogenesis (Cooper-Kuhn et al., 2004; Kaneko et al., 2006; Paez-Gonzalez et al., 2014; Asrican et al., 2016), a postnatal brain process that is required to maintain adequate olfactory discrimination of odorants (Moreno et al., 2009). Therefore, we hypothesized that the deficit in discrimination of perceptually similar odors in the M1/M3^−/−^ mice might result from a deficiency in the generation of adult-born neurons. We labeled the newly generated neurons by injecting the animals with BrdU, which is incorporated into the DNA of dividing cells. Accordingly, we quantified the density of BrdU+ neurons in the granule cell layer (GCL) of WT and M1/M3^−/−^ mice at 12 and 18 weeks postnatal (Figure 4A) using immunofluorescence against BrdU and NeuN (a marker of mature neurons, Figure 4B). As shown in Figure 4C, we found that M1/M3^−/−^ mice at 3 months of age show a significantly lower density of BrdU+ neurons in the GCL in comparison to the WT mice (2624 ± 429 vs. 5348 ± 723 cells/mm^3^; *n* = 4; *p* < 0.05). The total average density of adult generated neurons across the MOB of M1/M3^−/−^ mice was also significantly lower compared to the WT (1213 ± 87 vs. 2655 ± 234 cells/mm^3^; *p* < 0.05). This suggests that at this age, a decrease in the number of newborn neurons integrated in the OB in the KO mice could play an additional role in the olfactory deficits they exhibit.

Surprisingly, when we quantified BrdU+ neuron density at four and half months of age, we did not observe a difference in adult neurogenesis between M1/M3^−/−^ and WT mice (Figure 4C). As previously shown, adult neurogenesis decreased with age (Nunez-Parra et al., 2011); however, M1/M3^−/−^ mice display surprisingly similar levels of BrdU+ cells in the GCL in comparison to their WT counterparts (2084 ± 233 vs. 2196 ± 302 cells/mm^3^; *n* = 5). Together these findings suggest that cholinergic excitation plays a diminished role in regulating adult born neuron survival in mice at later ages. However, we note that the natural discrimination of closely related odors in M1/M3^−/−^ mice is age-independent, implying that adult-born neuron density in these mice is not closely linked with discrimination ability.

### Odor Enrichment Improves the Odor Deficits in the M1/M3^−/−^ Mice

Previous work has shown that OE is a potent stimulator of adult neurogenesis (Rochefort et al., 2002). Furthermore, OE is known to increase perceptual learning, whereby mice are able to naturally discriminate closely related odors that were not discriminated previously (Mandairon et al., 2006b; Escanilla et al., 2008). We therefore examined the possibility that OE, and increased adult neurogenesis, could compensate for the olfactory discrimination deficit observed in the M1/M3^−/−^ mice. To this extent, mice were exposed to different natural odors in their home-cage that were changed daily for a total period of 40 days. Three weeks into the enriched paradigm, animals were injected with BrdU and the number of OB-integrated neurons was quantified 4 weeks after injection (Figure 4A, bottom). As shown in Figure 4C, OE significantly increased the density of BrdU+ neurons in the GCL of both M1/M3^−/−^ (3600 ± 125 cells/mm^3^; *n* = 7) and WT mice (3596 ± 460 cells/mm^3^; *n* = 8), in comparison to mice exposed to empty odor containers (M1/M3^−/−^: 2084 ± 233 cells/mm^3^, *n* = 5; WT: 2196 ± 302 cells/mm^3^; *n* = 4; *p* < 0.05).

To assess the influence of OE on odor discrimination, we performed habituation-dishabituation tests on M1/M3^−/−^. Before the exposure, natural odors, M1/M3^−/−^ mice could not discriminate between C7-C8 odor pair (C7, 3.2 ± 0.7 s; C8, 3.3 ± 0.7 s; *n* = 13; *p* = 0.94; Figure 4D); however, after exposure to an odor-enriched environment these mice discriminated the odor pair (C7, 3.5 ± 0.5 s; C8, 9.0 ± 0.6 s; *n* = 6; *p* < 0.001). M1/M3^−/−^ mice exposed to empty odor containers still displayed a deficiency in naturally discriminating these odors (C7, 3.4 ± 0.7 s; C8, 4.1 ± 1.0 s; *n* = 5; *p* = 0.72, not shown).

These findings suggest that OE produces changes within the OB circuit necessary for the proper discrimination of perceptually similar odors. Additionally, because M1/M3^−/−^ mice reared in conditions with empty odor containers have similar levels of neurogenesis in comparison to their WT counterparts, but still fail to discriminate odors that differ by a single carbon, neurogenesis and odor discrimination may not be as tightly coupled as thought (Mandairon and Linster, 2009). In contrast they could be two alternative means to increase olfactory discrimination at different time-scales at later ages.

## Discussion

Previous work has shown a prominent role of mAChRs, in particular the M1 receptor, in regulating the excitability of OB neurons (Pressler et al., 2007; Smith and Araneda, 2010; Smith et al., 2015). Here, we show that transgenic KO mice lacking the M1 mAChR, the M1^−/−^ and the M1/3^−/−^ mice, exhibit impaired olfactory processing. Specifically, they showed altered short- and long-term olfactory memory and an age-dependent decrease in adult neurogenesis of GCs. However, this decrease in neurogenesis could be reversed by OE, which also improved perceptual learning, suggesting that these mechanisms are independent of M1-mediated cholinergic function.

ACh elicits its action by binding to both nicotinic and muscarinic receptors and a decrease in cholinergic function is associated with neurodegenerative diseases such as Alzheimer’s in which patients lose their sense of smell (Lucas-Meunier et al., 2003). In the olfactory system, activation of both receptor types has been shown to play a role in short and long-term odor memories (Fletcher and Chen, 2010) and sensory discrimination (Doty et al., 1999; Mandairon et al., 2006a; Chaudhury et al., 2009; Devore and Linster, 2012; Devore et al., 2012). The nicotinic receptors are mainly expressed in the most superficial layers of the OB (Le Jeune et al., 1995) where they regulate the input of information from the olfactory sensory neurons (Castillo et al., 1999; D’Souza and Vijayaraghavan, 2012; D’Souza et al., 2013). It has been postulated that this arrangement creates a filtering mechanism that increases the amount of neurotransmitter release required to successfully activate MCs, ultimately enhancing odor contrast (D’Souza and Vijayaraghavan, 2014).

The most abundant neurons in the OB are the inhibitory GCs, which regulate the excitability of the principal neurons, the MCs, through GABAergic inhibition (Shepherd, 2004). Muscarinic regulation of olfactory processing, in contrast to nicotinic modulation, appears to target the GC-MC dendrodendritic synapses in deeper layers of the OB. Activation of mAChR is thought to modulate the output of information from the OB to cortical regions (Pressler et al., 2007; Smith and Araneda, 2010; D’Souza and Vijayaraghavan, 2014). However, recent work has shown that cholinergic modulation of the MOB circuit is complex (Smith et al., 2015). MCs in the MOB, but not in the AOB, are inhibited by activation of M2 mAChR while they also exhibit a nAChR-mediated depolarization (Smith and Araneda, 2010; D’Souza and Vijayaraghavan, 2012; Smith et al., 2015). In contrast, GCs exhibit a M2-mediated hyperpolarization and an M1-mediated afterdepolarization. The overall, effect on cholinergic activation of MCs remains unknown, however, *in vitro* endogenous release of ACh by optogenetic activation of cholinergic afferents inhibited MCs, suggesting an overall decrease in MC activity (Smith and Araneda, 2010; Smith et al., 2015). Furthermore, M1 activation of GCs has been shown to promote MC inhibition in the MOB (Pressler et al., 2007). On the other hand, *in* *vivo* studies have shown that optogenetic stimulation from the basal forebrain sharpens the olfactory receptive fields of MCs, either by increasing odorant-mediated inhibition or excitation output neurons (Ma and Luo, 2012). Interestingly, when cholinergic release was optogenetically induced in the cholinergic terminals in the OB in an *in vivo* anesthetized mouse, there was an constant increase in excitability of MCs (Rothermel et al., 2014), suggesting that cholinergic modulation is complex and state-dependent. Moreover, using calcium imaging it was shown that ACh may have a dual action: increasing odor sensitivity to weak inputs through activation of M2-mAChRs and decreasing odor sensitivity to strong inputs trough nAChRs (Bendahmane et al., 2016). The same study found that M1 receptors did not play a significant role increasing glomerular activation and odor sensitivity. In agreement, we did not find any difference in the odor detection threshold of the M1/M3^−/−^ mice.

In agreement with an M1 excitatory effect on GCs of MOB, we found that the M1^−/−^ and M1/M3^−/−^ mice showed an impairment of odor perceptual learning. These mice failed to discriminate between chemically similar odorants, such as odors differing by one carbon moiety or stereoisomers, in a habituation-dishabituation test. These findings are also in agreement with previous *in vivo* studies that utilized a pharmacological approach, where disruption of muscarinic response in the OB decreased odor discrimination (Chaudhury et al., 2009; Devore et al., 2012). Nevertheless, the impairment in perceptual learning was not extensive as the M1/M3^−/−^ mice could naturally discriminate between more dissimilar odors, such as odors differing by two-carbon moiety and social odors. This limited impairment in the M1/M3^−/−^ mice is in agreement with previous studies showing that disruption of cholinergic function in the OB results in generalization of similar, but not dissimilar odors (Linster et al., 2001). Interestingly, despite the compensatory mechanism observed in the M1^−/−^ KO that lead us to the use of the M1/M3^−/−^ mice, both lines exhibited a very similar olfactory behavior phenotype further suggesting that absence of the M1 mAChR underlies the olfactory impairment.

Previous studies have suggested that cholinergic modulation, specifically through muscarinic receptors, plays an important role in long-term odor learning (De Rosa and Hasselmo, 2000; Saar et al., 2001). Interestingly, mice lacking the M1 and M3 mAChR were able to learn the instrumental aspect of an associative-odor learning task, although at a significantly slower rate than compared to that of the WT mice. Repetitive exposure of an odorant, increases the capacity of rodents to discriminate them (Fletcher and Wilson, 2002) by changing the receptive field of MC in the OB (Fletcher and Wilson, 2003) and enhancing the salient odor. Accordingly, after 2 days of exposure to the carvone isomers during associative training WT mice could discriminate the isomers in a habituation-dishabituation test. Olfactory perceptual learning depends on muscarinic modulation (Fletcher and Wilson, 2002), yet the receptor isoforms involved in the process had not been described. Our data suggest that the M1 and M3 mAchR can, at least in part, mediate this modulation, since the M1/M3^−/−^ only discriminate between the carvone enantiomers after 4 days of associative training. Thus, the M1/M3^−/−^ mice show a significant impairment in perceptual odor learning due to repeated odor exposure, suggesting that an olfactory processing deficit could underlie their altered olfactory associative memory.

It should be noted that cholinergic modulation may also occur at the level of the olfactory cortex (OC), where the absence of M1 and M3 mAChRs could also disrupt olfactory processing. For instance scopolamine injection into the anterior OC produces a generalization or cross-habituation between odor representations (Wilson, 2001) possibly by reducing the inhibitory effect of ACh in the intrinsic circuit of the OC, potentiating the afferent MC-OC synapses (Hasselmo and Bower, 1992). Also, the M1/M3^−/−^ line used in our experiments is a constitutive KO, and M1-like mAChRs are expressed prominently throughout the brain; therefore, we cannot rule out the possibility that other brain functions are compromised (see Wess, 2004). For instance, it has been suggested that the basolateral amygdala plays an important role in associative memory and olfactory discrimination, specifically the motivational salience associated with a particular odor in a go-no go task (Schoenbaum et al., 1999). Moreover muscarinic modulation through M1 receptors regulates the excitability of the neurons in this brain region (Unal et al., 2015). Further studies with conditional KO in specific components of the OB circuit could address this issue.

Interestingly, we found an age-dependent cholinergic regulation of neurogenesis. Our results indicated that M1M3^−/−^ mice display lower levels of neurogenesis at 3-months of age. We hypothesize that this decrease in adult neurogenesis could arise from the diminished cholinergic signaling since it has been suggested that ACh plays a critical role in the proliferation (Cooper-Kuhn et al., 2004) and survival (Kaneko et al., 2006) of adult-generated neurons in the OB and other neurogenic niches such as the subgranular zone of the hippocampus. Interestingly, the differences in the number of adult-generated neurons in the OB of WT and M1/M3^−/−^ was not maintained at older ages, where both strains showed a similar age-dependent decline in adult neurogenesis. This suggests that the role of cholinergic excitation in regulating neurogenesis occurs in an age-dependent manner. Adult neurogenesis exhibits a dramatic age-dependent decrease in the OB, specifically after 2 months (Ahlenius et al., 2009; Enwere et al., 2004; Nunez-Parra et al., 2011). Thus, it is possible that the cholinergic system could exert a stronger regulation only in younger mice.

Both young and older M1/M3^−/−^ mice exhibit impaired olfactory discrimination, therefore our finding that the deficit in neurogenesis is not present in older M1/M3^−/−^ mice was unexpected. Several studies emphasize the necessity of adult neurogenesis of GCs in olfactory discrimination (Rochefort et al., 2002; Alonso et al., 2006; Bovetti et al., 2009; Moreno et al., 2009). However, our findings and results from other groups suggest that adult neurogenesis and the ability to distinguish similar odors may not be so tightly coupled (Saghatelyan et al., 2005; Valley et al., 2009; Sakamoto et al., 2014). Instead, a lack of excitatory cholinergic function in preexisting GCs of the OB may be preventing the necessary computations that give rise to the ability to distinguish similar odors. This idea is supported by experiments that show the effects of muscarinic antagonism in blocking odor discrimination (Mandairon et al., 2006a; Chaudhury et al., 2009; Chapuis and Wilson, 2013). This is also in agreement with our observation that WT mice with an intact cholinergic system, showed an improvement in perceptual learning during the training trials in the associative learning within a day.

Lastly, previous work has revealed the strong influence of an olfactory-enriched environment on adult-born neuron survival (Bovetti et al., 2009) and perceptual learning and olfactory discrimination (Rochefort et al., 2002; Moreno et al., 2009; Veyrac et al., 2009). In agreement with these studies, we observed a significant increase in adult-born neuron density in mice exposed to enriched conditions. However, our findings suggest that adult born cell survival under enriched conditions do not rely on an M1/M3 excitatory cholinergic effects as mice lacking these receptors display similar increases in neurogenesis as compared to WT mice. Additionally, increased levels of adult-born neuron in the M1/M3^−/−^ mice after enrichment are accompanied by an acquired ability to distinguish odors that differ by a single carbon. Our results suggest the improvement in perceptual learning produced by OE is independent of M1 mediated excitatory actions on GCs, or M1/M3 mAChRs elsewhere.

In summary, muscarinic regulation mediated by M1 and M3 mAChRs is critical to elicit adequate olfactory-mediated behaviors that rely on both, short and long-term memory. Moreover, our results suggest that the olfactory system exhibits two complementary plastic mechanisms to increase the salience of odorants: a fast and transient muscarinic neuromodulations and a slower, activity-dependent and long-lasting integration of inhibitory neurons in the adult brain.

## Author Contributions

WC, SS, TK, RD, CE and RM conducted experiments and analyzed data. WC, AN-P and RCA wrote the manuscript. RCA designed the experiments.

## Conflict of Interest Statement

The authors declare that the research was conducted in the absence of any commercial or financial relationships that could be construed as a potential conflict of interest.

## Footnotes

^1^http://www.gpower.hhu.de/en.html
